# IDPsBind: a repository of binding sites for intrinsically disordered proteins complexes with known 3D structures

**DOI:** 10.1186/s12860-022-00434-5

**Published:** 2022-07-26

**Authors:** CanZhuang Sun, YongE Feng, GuoLiang Fan

**Affiliations:** 1 College of Science, Inner Mongolia Agriculture University, Hohhot, 010018 People’s Republic of China; 2 Department of Physics, School of Physical Science and Technology, Inner Mongolia University, Hohhot, 010020 People’s Republic of China

**Keywords:** Intrinsically Disordered Proteins, Intrinsically Disordered Proteins Complexes, Binding sites, PDB

## Abstract

**Background:**

Intrinsically disordered proteins (IDPs) lack a stable three-dimensional structure under physiological conditions but play crucial roles in many biological processes. Intrinsically disordered proteins perform various biological functions by interacting with other ligands.

**Results:**

Here, we present a database, IDPsBind, which displays interacting sites between IDPs and interacting ligands by using the distance threshold method in known 3D structure IDPs complexes from the PDB database. IDPsBind contains 9626 IDPs complexes and 880 intrinsically disordered proteins verified by experiments. The current release of the IDPsBind database is defined as version 1.0. IDPsBind is freely accessible at http://www.s-bioinformatics.cn/idpsbind/home/.

**Conclusions:**

IDPsBind provides more comprehensive interaction sites for IDPs complexes of known 3D structures. It can not only help the subsequent studies of the interaction mechanism of intrinsically disordered proteins but also provides a suitable background for developing the algorithms for predicting the interaction sites of intrinsically disordered proteins.

## Background

Intrinsically disordered proteins (IDPs) lack a stable secondary or tertiary structure under physiological conditions. Still, they participate in many important biological processes such as cell signal transduction, DNA metabolism, mRNA alternative splicing, protein–protein interaction, and so on [1–3]. Recent studies have shown that IDPs are associated with some diseases when modifications, translations, or expressions of IDPs are abnormal [4–6]. Due to the importance of IDPs in organisms, IDPs have become a hot spot in the current research on protein function. Over the past 20 years, many IDPs have been validated experimentally or computationally. Disprot is the first curated database containing a collection of experimentally validated IDPs and IDP disordered regions. [7]. The Disprot database includes a total of 1590 IDPs sequences, excluding the ambiguous and obsolete regions in release 2020_12. These IDPs are from 10 different species. The D^2^P^2^ database consists of computationally predicted IDPs from distinct proteomes [8], in which annotations for IDPs are derived from MobiDB. MobiDB3.0 [9] provides information about intrinsically disordered regions (IDRs), related features from various sources, and prediction tools. Different levels of reliability and different features are reported as different and independent annotations. The IDEAL [10] is a database incorporating functional with structural/disorder annotations for IDPs by manually integrating protein databank (PDB) [11].

These IDPs perform critical biological functions by interacting with other proteins or ligands. Currently, there are some databases about binding sites [12–15], such as Disbind, in which contains 226 IDPs with functional site annotations and binding ligands, including proteins, RNA, DNA, metal ions and others, respectively. However, studying IDPs-ligand interactions is still challenging due to the flexible binding affinity. Lack of enough information on how IDPs interact with other molecules, the biological functions for mostly IDPs are unknown. Although existing databases contain some helpful information, the number of IDPs and IDPs complexes is too tiny to support further study of the IDPs-ligand interaction. With the progress of structural biology, the number of protein structure files in PDB is growing rapidly, among which the structure files of IDPS are also increasing. This motivates us to develop a comprehensive IDPs-ligand interaction database and provide more interacting sites. In this paper, we introduced the IDPsBind database, which contains 9626 IDPs complex interactions. IDPsBind displays binding sites between IDPs and interacting ligands by using the distance threshold method in known 3D structure IDPs complexes from the PDB database. IDPs are selected from the Disprot database (release 2020_12). Each entry in IDPsBind contains a comprehensive list of annotations: primary information of an IDPs, sequence information, and binding sites information in PDB sequences.

## IDPsBind database construction

The following is the procedure for IDPsBind construction. (a) The intrinsically disordered proteins (IDPs) are derived from the Disprot (http://www.Disprot.org/, release: 2020_12) [7]; (b) Eliminate those IDPs with ambiguous and obsolete regions; (c) IDPs-ligand complex structures with x-ray crystallography resolution of better than 3.5 angstroms in the PDB Database are selected for study; (d) Elimination those IDPs with mutant residues in the IDPs complexes; (e) Binding ligands of IDPs are selected from HETATM in the PDB file. Finally, IDPsBind contains 880 IDPs and 9626 IDPs complexes (from PDB).

**Fig. 1:**
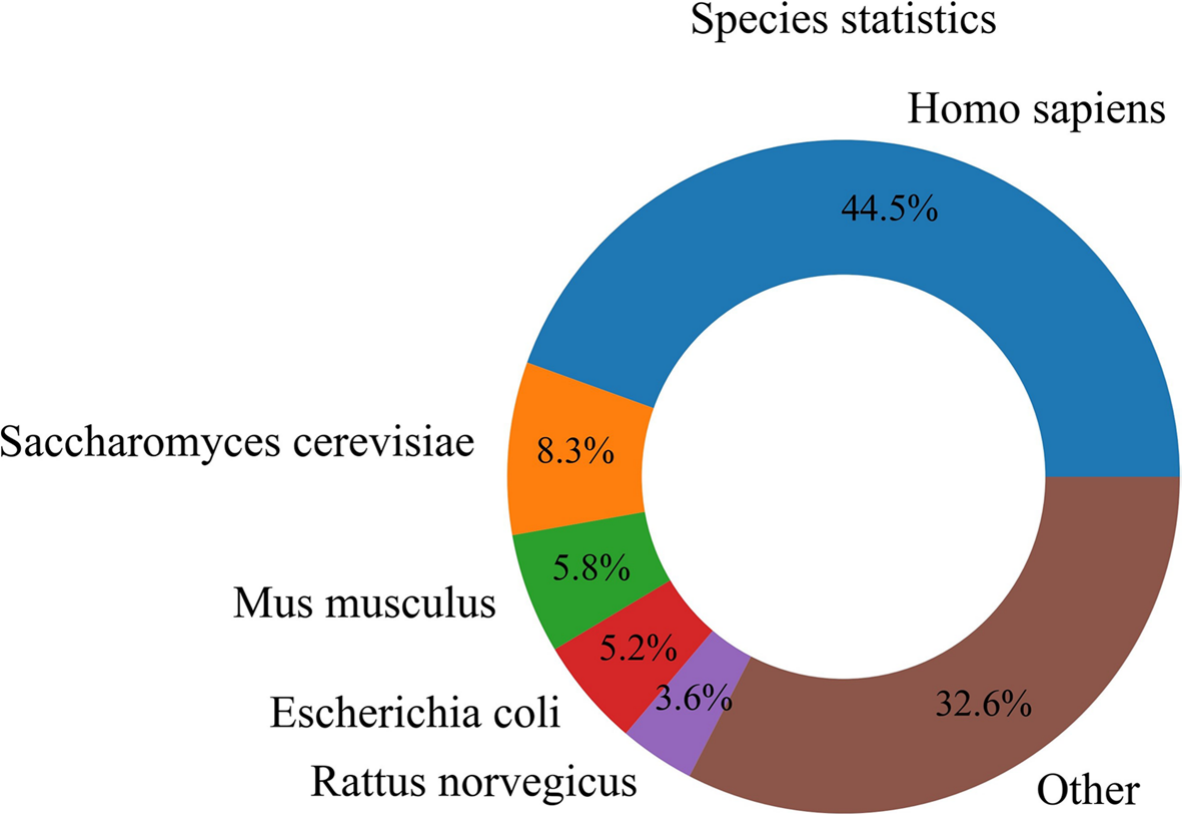
Distribution of 880 IDPs in the organism

**Fig. 2:**
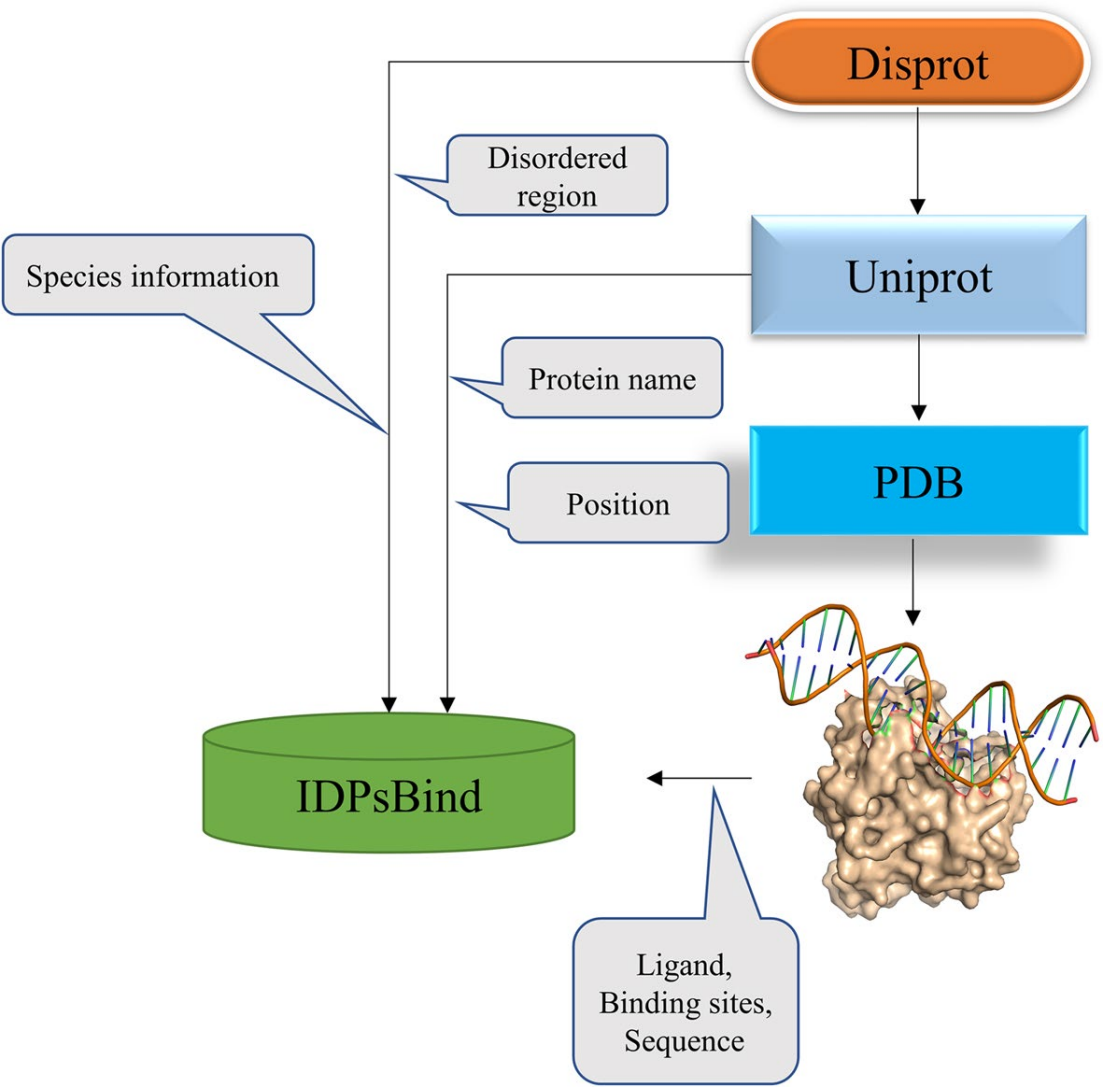
Workflow of the construction of IDPsBind

**Fig. 3:**
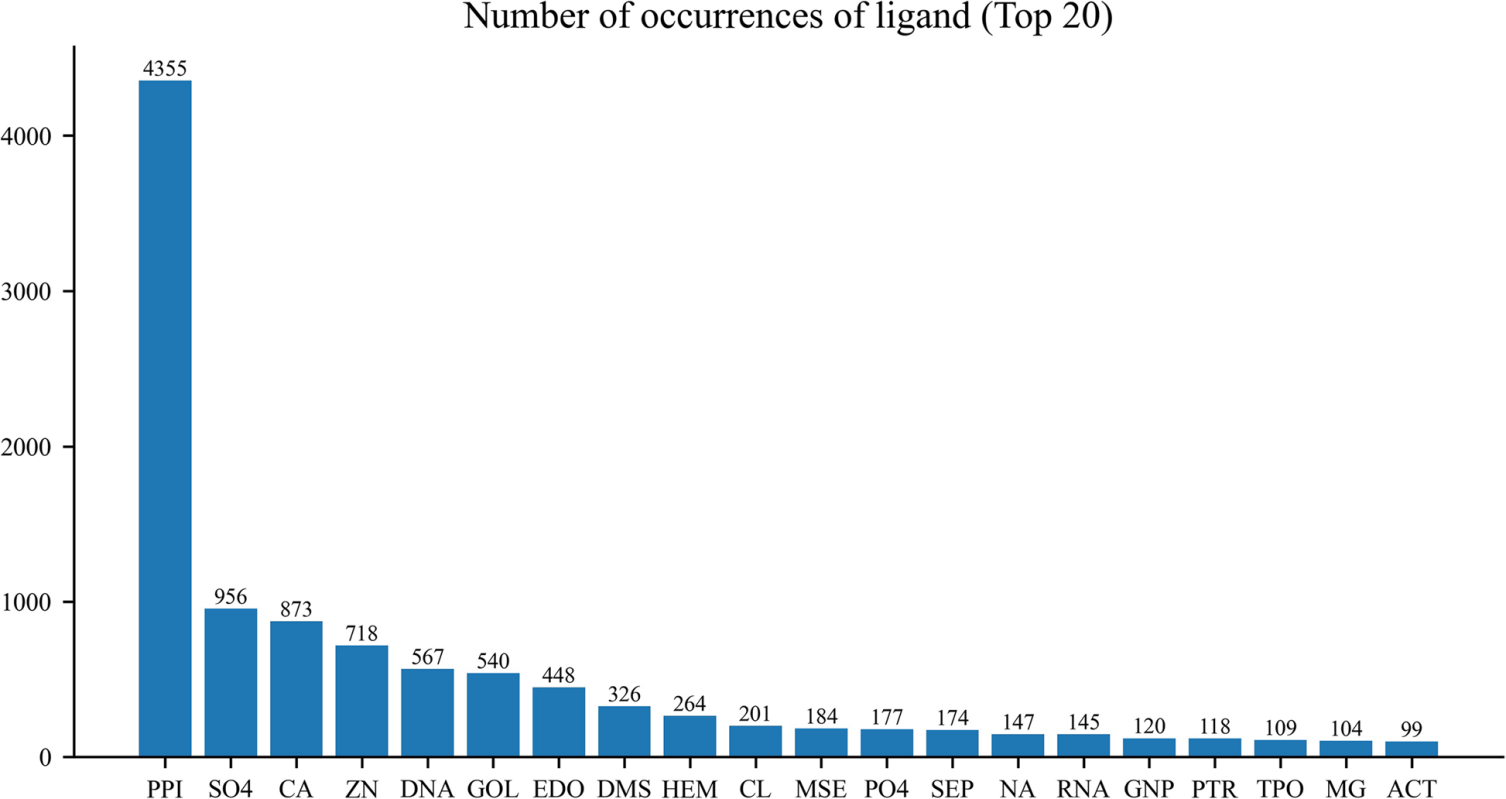
Distribution of the top 20 binding ligands

**Fig. 4:**
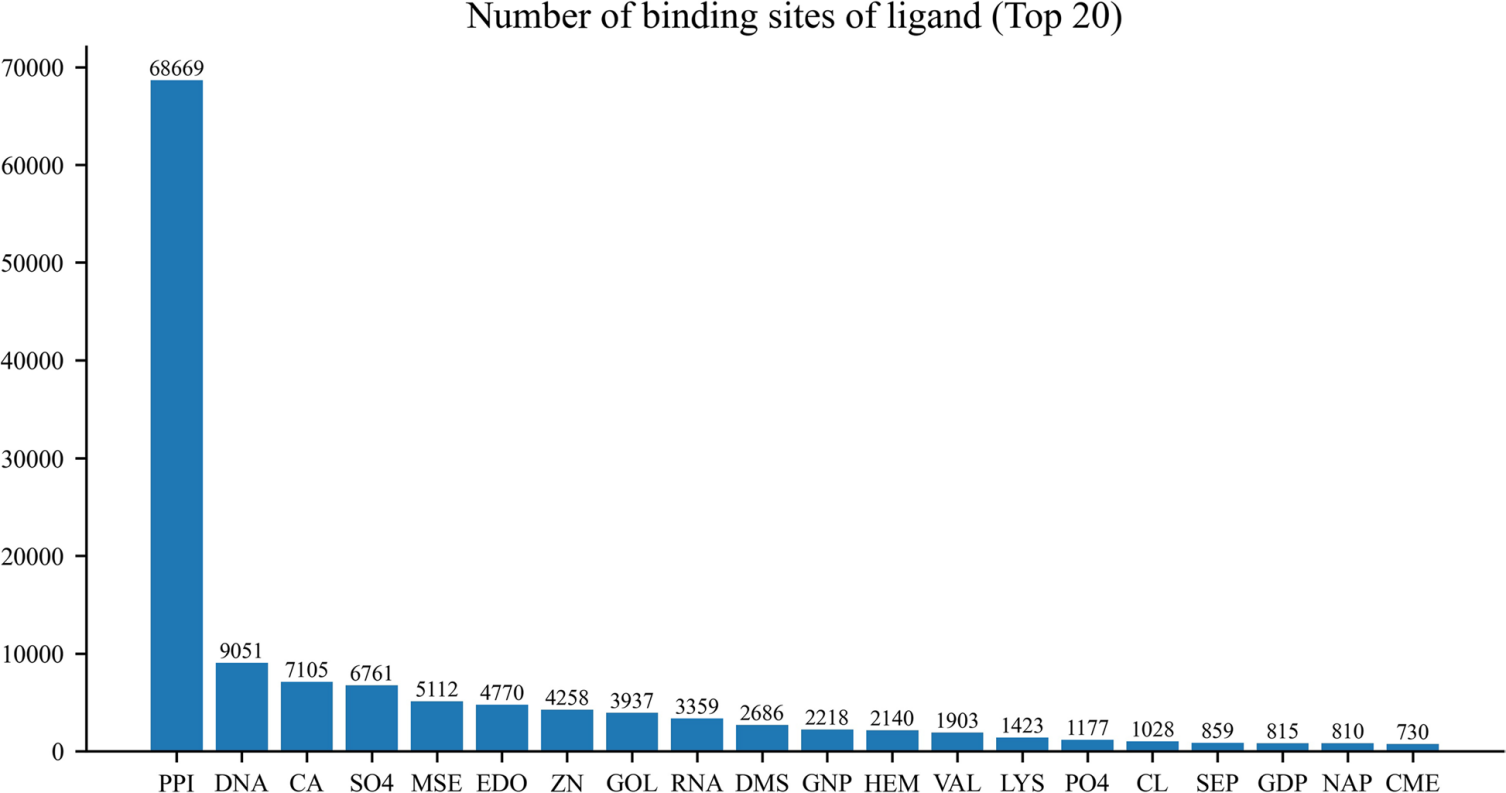
Distribution of the top 20 most numerous binding sites

As in previous studies, an amino acid residue within a protein sequence is designated as a binding site if it contains at least one atom that falls within a cutoff distance from any atoms of the ligand molecule in the complex [16–19]. Binding residues in IDPsBind are determined by a distance cutoff of 3.5 angstroms between any atoms of a protein. All corresponding PDB chains (resolution better than 3.5 angstroms) for an IDP are used for analysis. All binding ligands and binding sites information are derived from ATOM and HETATM in the PDB file. The construction process of the IDPsBind database, the distribution of binding ligands and binding sites in the IDPsBind, are shown in Figs. 1, 2, 3 and 4.

## Web interface

IDPsBind provides six basic interfaces: Home, Browse, Download, Search, Statistics, and Help. The ‘**Home**’ page describes the introduction of the IDPsBind database provides links to the three primary associated databases. The ‘**Browse**’ page displays the summary of all entries in the IDPsBind database. This interface lists six components: IDPsBind ID, Disprot ID, UniProt ID, Protein name, Source, and Disordered content. All items collected in IDPsBind numbered from IDP0001 to IDP0880 can be retrieved by clicking the ‘**Browse**’ option. Clicking any IDPsBind ID will return a display of the detailed information on the target chain. The data stored for each ID has three parts. The first part shows the basic information about the protein. The second part provides the sequence of IDPs in the Disprot database and color-codes the disordered regions. The third part shows the labeled binding sites of the PDB chains (resolution better than 3.5 angstroms) when the protein interacts with ligands. Moreover, the interface displays the abbreviation of the ligand, click the **‘?’** label will show the full name of the ligand. Users can also check specific ligand information clicking the abbreviated ligand jump link to a new interface. And clicking the ‘**load**’ option, the corresponding structure of the complex is visualized on the right side of the page. The IDPsBind database is freely available for download. Users can download them as a whole on the ‘**Download**’ page. On the **Search** interface, users enter any keyword or IDPsBind /Disprot / Uniprot ID and then click the ‘search’, then the page shows the results similar to the “**Browse the entry**” page. The ‘**Statistics**’ interface shows some basic data and information about the IDPsBind database. The ‘**Help**’ interface answers some questions on the IDPsBind database. The current release of IDPsBind is 1.0, which will be updated in the future during the PDB release update.

## Conclusions

We have developed a comprehensive IDPs-ligand interaction database, IDPsBind, in which IDPs are taken from the DisProt database (2020_12), and corresponding IDPs complexes are from the PDB database. Although there are already a handful of ligand-binding databases in the literature, IDPsBind is distinguished from other databases in the following aspects. (a) IDPsBind contains many interactions of IDPs, 3203 binding ligands including proteins, DNA, RNA, et al. (b) The interaction includes not only the disordered regions with the ligand, but also that of the ordered regions in IDPsBind. (c) The IDPs-ligand binding information is based on the PDB file, and all the PDB chains (resolution better than 3.5 angstroms) for IDPs were analyzed. In this way, ligand-binding sites of the target chain cannot be missing in IDPsBind. (d) All data in IDPsBind database are freely available for download. We hope that the IDPsBind can provide helpful information required for specific IDPs-relevant studies.

## Data Availability

The author can provide compiled executable file on data in this article. Please send an email to the author (yefeng@imau.edu.cn) to query the relevant data of this paper. And all data can be downloaded freely in IDPsBind.
